# Shoot organogenesis and somatic embryogenesis from leaf and root explants of *Scaevola* *sericea*

**DOI:** 10.1038/s41598-020-68084-1

**Published:** 2020-07-09

**Authors:** Hanzhi Liang, Yuping Xiong, Beiyi Guo, Haifeng Yan, Shuguang Jian, Hai Ren, Xinhua Zhang, Yuan Li, Songjun Zeng, Kunlin Wu, Feng Zheng, Jaime A. Teixeira da Silva, Youhua Xiong, Guohua Ma

**Affiliations:** 1 Guangdong Provincial Key Laboratory of Applied Botany and Engineering Laboratory for Vegetation Ecosystem Restoration On Islands and Coastal Zones, South China Botanical Garden, The Chinese Academy of Sciences, Guangzhou, 510650 China; 2 College of Horticulture and Landscape Architecture, Zhongkai University of Agriculture and Engineering, Guangzhou, 510225 China; 3 Miki-cho Post Office, P.O. Box 7, Ikenobe 3011-2, Miki-cho, Kagawa-ken 761-0799 Japan; 4 University of Chinese Academy of Sciences, Beijing, 100039 China; 5 Cash Crop Institute of Guangxi Academy of Agricultural Sciences, Nanning, China

**Keywords:** Biotechnology, Cell biology, Developmental biology, Plant sciences

## Abstract

An efficient regeneration system via shoot organogenesis and somatic embryogenesis from in vitro leaf and root explants was established for *Scaevola sericea* for the first time. The highest axillary shoot proliferation coefficient (4.8) was obtained on Murashige and Skoog (MS) medium supplemented with 1.0 mg/L 6-benzyladenine (BA) and 0.1 mg/L *α*-naphthaleneacetic acid (NAA) every 45 days. Young in vitro leaves and roots, which were used as explants, were cultured onto medium supplemented with different plant growth regulators. Our results showed that only cytokinins BA and thidiazuron (TDZ), could induce adventitious shoots and somatic embryos from leaf and root explants. The optimal medium to achieve this was MS medium supplemented with 2.5 mg/L BA and which induced most adventitious shoots (2.7) and somatic embryos (17.3) from leaf explants within 30 days. From root explants, 1.1 adventitious shoots and 7.6 somatic embryos could be induced on MS medium supplemented with 2.5 mg/L TDZ. Histological observation showed that both somatic embryos and adventitious shoots were originated from homogeneous parenchyma and the development of somatic embryos was visible. Maximum rooting percentage (99.0%) was achieved on half-strength MS medium supplemented with 2.5 mg/L NAA. Well-rooted plantlets, which were transplanted into a substrate of pure river sand, displayed a high survival percentage of 91.7% after transplanting for 45 days while the best substrate for plantlet growth was river sand: coral sand (1:1).

## Introduction

*Scaevola* *sericea* Vahl (syn. *Scaevola taccada* (Gaertn.) Roxb.), commonly known as beach naupaka, is a semi-mangrove tree from the Goodeniaceae that consists of 13 genera with 329 accepted species^1^, and almost all the original and existing species are concentrated in Australia^2^. Only the genus *Scaevola* is widespread throughout the world^3^. There are a total of 300 scientific plant names of species rank for the genus *Scaevola*, 40 of which are distributed outside Australia, mostly in tropical island areas^4^. *S.* *sericea* is a dominant plant at the leading edge of beach communities throughout much of the tropical Pacific^5,6^. *S. sericea* has beautiful flowers with a long flowering period, beautiful branch type and leaves, so it is often bred as an ornamental and landscape plant^7,8^. In Hawaii, *S. sericea* is reputedly used to treat deep cuts, cataracts, scaly skin and punctures^9^. *S. sericea* also showed antiviral activity against herpes simplex virus-1 and 2 and vesicular stomatitis virus and also displayed some anti-fungal activity^10^. The leaves of *S. sericea* are rich in allopurinol, ^1^H-indole-3-acetic acid, hydrazide, ^2^H-1-benzopyran-2-one, 7- (ethylamino)- 4,6- dimethyl, *n*-hexadecanoic acid, scopoletin, 9,12,15-octadecatrienoic acid, 7-hydroxycoumarin and other chemical components that offer protection against *Proteus vulgaris*, *Staphylococcus aureus*, *Pneumonia bacteria* and *Pseudomonas aeruginosa*^11,12^. In vivo anticancer activity against Ehrlich ascites carcinoma in Swiss albino mice was also assessed from leaf extracts^13^. The conventional reproduction of *S. sericea* is by seed. However, seed reproductive ability is poor, and seed germination percentage in the genus *Scaevola* is low and takes a long time^2^. To date, no report exists on shoot organogenesis and somatic embryogenesis of *S. sericea*. In this study, on the base of primarily establishment of shoot proliferation of *S. sericea* as a halophyte species, we also comparing NaCl-induced stress physiological responses of six halophyte species in *in vitro* and *in vivo* culture^14^. An efficient regeneration system via shoot organogenesis and somatic embryogenesis from in vitro leaves and root explants was established in *S. sericea* for the first time.

## Results

### Axillary shoot proliferation

**Figure 1:**
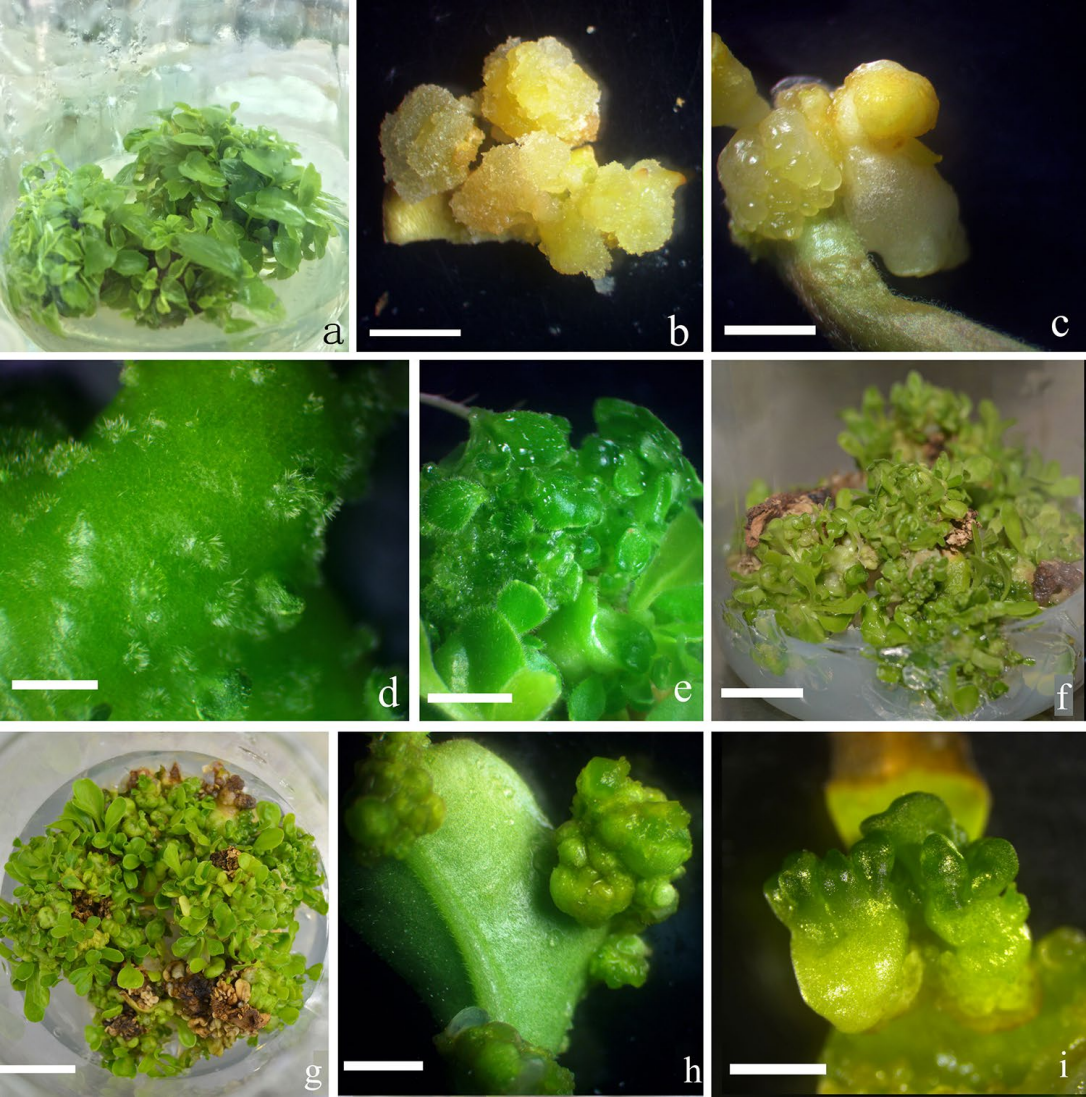
Adventitious shoot formation and somatic embryogenesis from leaf explants of *Scaevola sericea.* Bars = 2 mm. (**a**) Axillary shoots were proliferated on MS medium with 1.0 mg/L BA and 0.1 mg/L NAA; (**b**) light-cultured friable callus induced on MS medium with 0.5 mg/L 2,4-D; (**c**) dark-cultured globular callus clump induced on MS medium with 0.5 mg/L BA for 30 days; (**d**, **e**) light-cultured adventitious shoots induced on MS medium with 0.5 mg/L BA for 30 and 40 days, respectively; (**f**, **g**) light-cultured adventitious shoots induced on MS medium with 0.5 mg/L BA for 60 days (side and top views of jars, respectively); (**h)** light-cultured callus clump induced on MS medium with 0.5 mg/L zeatin for 30 days; (**i**) light-cultured somatic embryos induced on MS medium with 0.5 mg/L TDZ for 40 days.

Cytokinins had a positive effect on axillary bud proliferation, and the proliferation coefficient of axillary shoots increased as cytokinins concentration increased. The effect of 6-benzyladenine (BA) is greater than that of 6-Furfurylaminopurine (kinetin, KIN) (Table 1). The highest proliferation coefficient was 4.8 with 1.0 mg/L BA + 0.1 mg/L NAA, which was insignificantly different to the use of BA alone in MS medium at 1.5 mg/L, giving a proliferation coefficient of 4.4 (Fig. 1a).

**Table 1:** Effect of plant growth regulators (PGRs) on axillary shoot proliferation of *Scaevola sericea* after culture for 30 days.

PGRs (mg/L)	Shoot proliferation coefficient (SPC)
BA 0.1	2.5 ± 0.3 d
BA 0.5	3.3 ± 0.2 c
BA 1.5	4.4 ± 0.2 ab
KIN 0.1	2.2 ± 0.2 d
KIN 0.5	2.4 ± 0.2 d
KIN 1.5	3.1 ± 0.2 c
BA 0.1 + NAA 1.0	3.9 ± 0.3 b
BA 0.5 + NAA 0.5	4.1 ± 0.3 b
BA 1.0 + NAA 0.1	4.8 ± 0.3 a

Each treatment had 50 shoots. Different letters within a column indicate significant differences according to Duncan’s multiple range test (*P* < 0.05).

### Induction of shoots and somatic embryos from leaf explants

When leaf explants were light cultured on medium containing 0.5–2.5 mg/L 2,4-dichlorophenoxyacetic acid (2,4-D) for 30 days, they became swollen and induced yellow friable callus (Fig. 1b), but on medium with 0.5–2.5 mg/L *α*-naphthaleneacetic acid (NAA) for the same period, some callus was induced at the cut (wounded) section. A few adventitious roots developed from the callus. However, no somatic embryo or adventitious shoot was visible within 30–60 days.

On MS medium with 0.5 mg/L BA and culture in the dark for 30 days, yellow compact callus was induced, developing some globular somatic embryos (Fig. 1c). As cultures were transferred to light, leaf explants became green and some protuberances developed on the leaf surface (Fig. 1d) that developed some adventitious shoots within 30 days (Fig. 1e).

**Figure 2:**
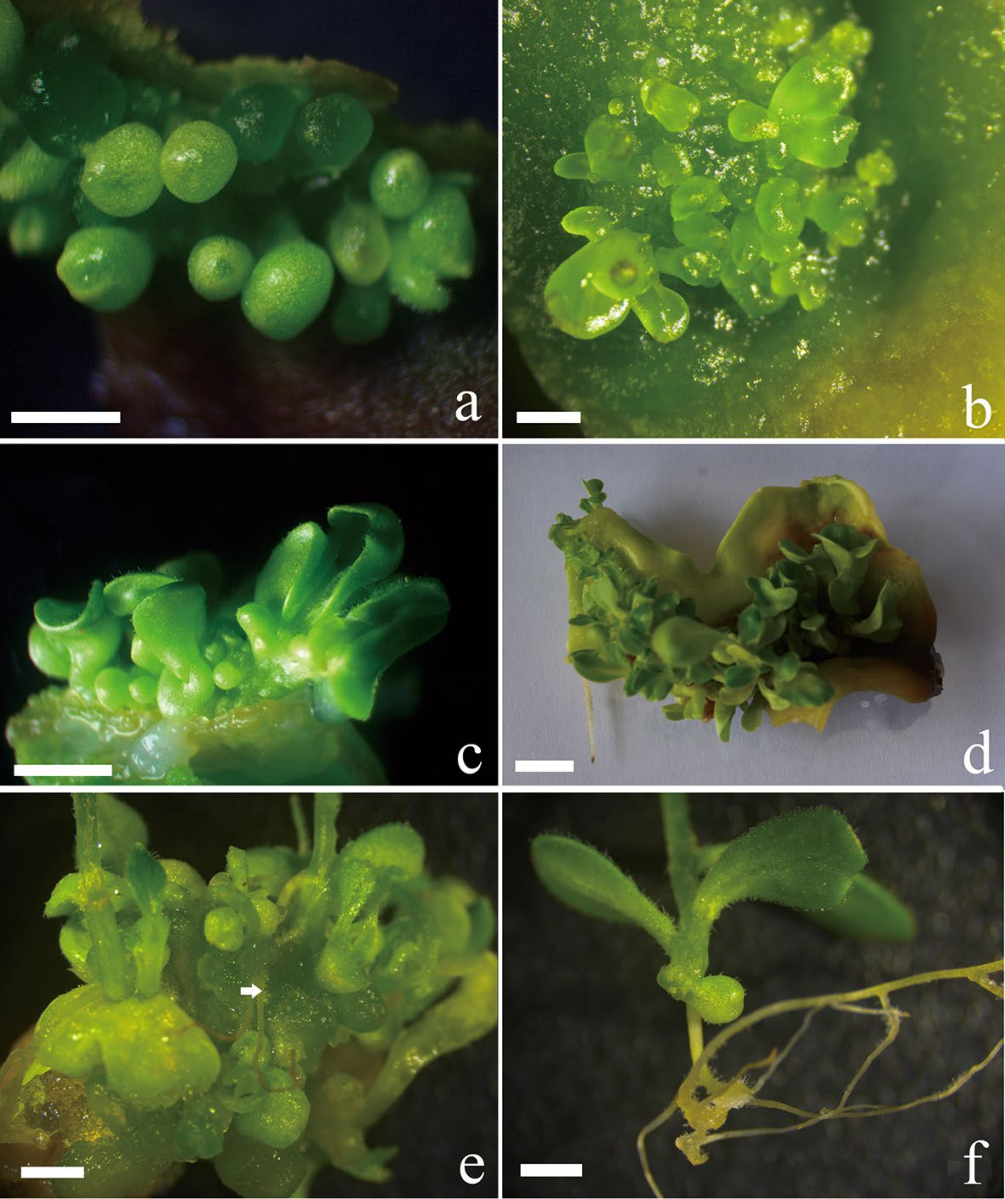
Somatic embryogenesis from leaf explants of *Scaevola sericea.* Bars = 2 mm. (**a**) Globular somatic embryos were induced after culture for 30 days on MS medium with 2.5 mg/L BA; (**b**) somatic embryos were induced after culture for 40 days on MS medium with 1.0 mg/L BA; (**c**, **d**) somatic embryos were induced after culture for 40–50 days on MS medium with 2.5 and 5.0 mg/L BA, respectively; (**e**) somatic embryos were induced after culture for 60 days on MS medium supplemented with 2.5 mg/L BA showing radicle and root development (white arrow); (**f**) a single somatic embryo, which was isolated directly from the clump (**e**), developed into a plantlet.

Increasing BA concentration to 2.5 mg/L formed more globular somatic embryos and fewer adventitious shoots on the leaf surface within 30 days (Fig. 2a). When culture period was prolonged to 40 days, globular somatic embryos became heart- and cotyledon-shaped somatic embryos formed within 40–50 days (Fig. 2b–d). After further culture for 50–60 days, some somatic embryos developed a radicle and roots (Fig. 2e,f). As BA concentration increased, the total number of somatic embryos generally increased, most forming on MS medium with 2.5 mg/L BA within 30 days (Table 2). By prolonging culture period, a single jar with MS medium supplemented with 0.5 mg/L BA could induce hundreds of adventitious shoots and somatic embryos (Fig. 1f,g).

**Table 2:** Effects of plant growth regulators (PGRs) on induction of adventitious shoots and somatic embryos from leaf explants of *Scaevola sericea* after light culture for 30 days.

PGRs (mg/L)	Number of adventitious shoots/ leaf	Number of somatic embryos/ leaf
2,4-D 0.1–2.5	0.0 ± 0.0 e	0.0 ± 0.0 e
NAA 0.1–2.5	0.0 ± 0.0 e	0.0 ± 0.0 e
BA 0.1	5.9 ± 0.5 b	0.0 ± 0.0 e
BA 0.5	5.3 ± 1.3 b	0.0 ± 0.0 e
BA 2.5	2.7 ± 0.9 c	17.3 ± 1.5 a
TDZ 0.1	2.8 ± 1.1 c	3.5 ± 0.4 d
TDZ 0.5	2.1 ± 1.1 cd	4.5 ± 0.6 d
TDZ 2.5	1.1 ± 0.5 d	8.5 ± 0.3 b
Zeatin 0.1	1.1 ± 0.3 cd	0 ± 0.0 e
Zeatin 0.5	2.1 ± 0.3 cd	0 ± 0.0 e
Zeatin 2.5	3.4 ± 0.3 cd	0 ± 0.0 e
BA 0.5 + NAA 0.1	6.3 ± 0.8 b	7.8 ± 1.4 b
BA 1.0 + NAA 0.5	8.2 ± 0.1 a	6.3 ± 0.8 c

Each treatment had 30 leaf explants. Different letters within a column indicate significant differences according to Duncan’s multiple range test (*P* < 0.05).

On MS medium with zeatin, only a few protuberances formed at cut sections or on the leaf surface (Fig. 1h). Few adventitious shoots developed in light culture (Table 2). No somatic embryo was found even zeatin concentration increased to 2.5 mg/L.

On MS medium with thidiazuron (TDZ), some leaf explants easily induced callus but fewer adventitious shoots and somatic embryos were induced than on medium with BA in light culture (Table 2). Some secondary somatic embryos developed on initial somatic embryos within 50–60 days (Fig. 1i).

The use of both BA and NAA in MS medium induced more adventitious shoots and somatic embryos than when BA was used alone (Table 2).

### Induction of shoots and somatic embryos from root explants

**Figure 3:**
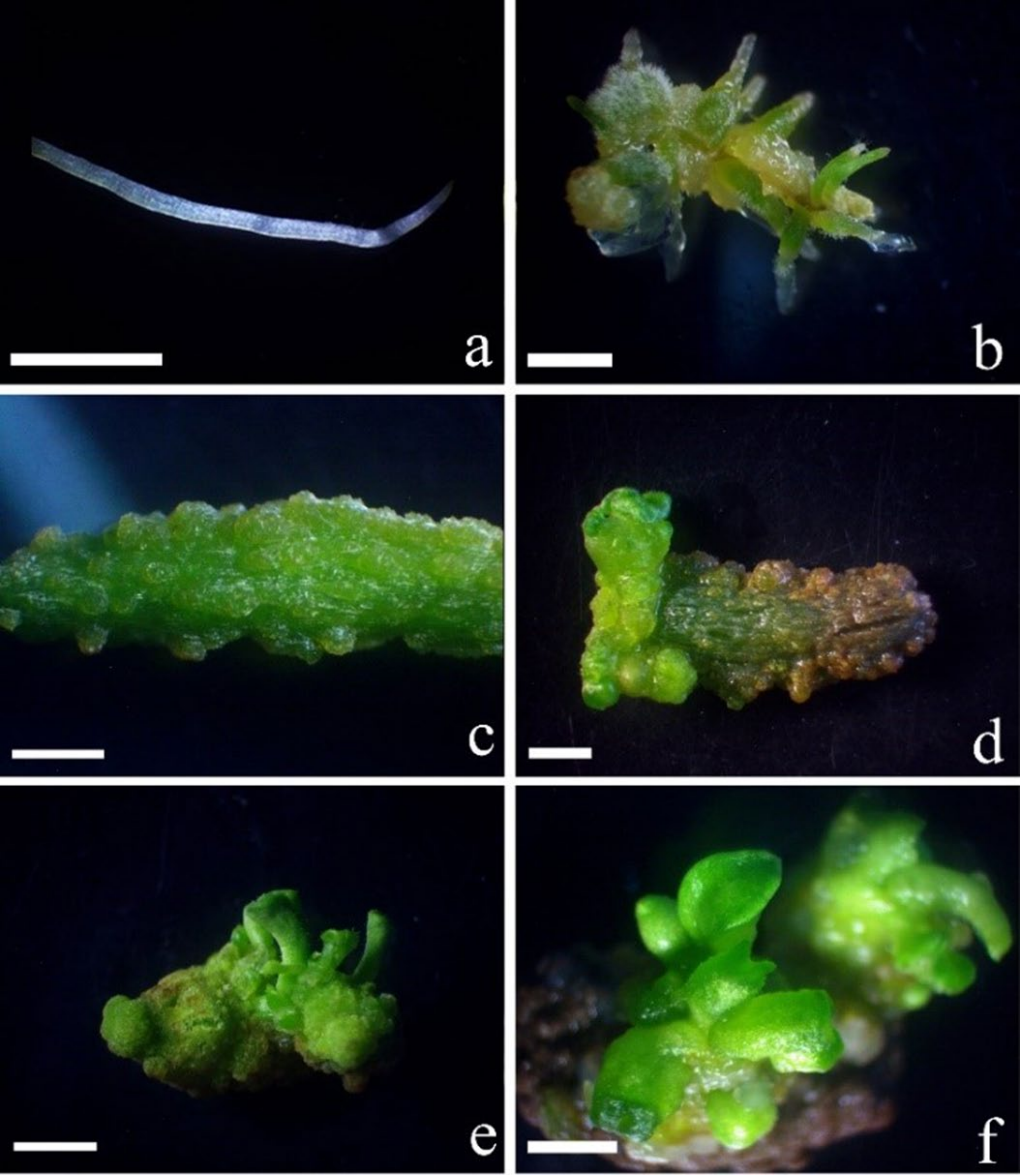
Somatic embryogenesis and shoot organogenesis from root explants of *Scaevola sericea*. (**a**) White root from plantlets cultured on MS medium with 0.5 mg/L NAA for 30 days; (**b**) roots became green and formed some adventitious roots after culture on MS medium with 2.5 mg/L NAA for 30 days; (**c**–**e**) roots cultured on MS medium with 2.5 mg/L BA induced some granular protuberances, globular- and heart-shaped somatic embryos after culture for 30, 40, and 50 days, respectively; (**f**) somatic embryos and adventitious shoots were induced on MS medium with 2.5 mg/L TDZ for 60 days. Bars = 2 mm.

White roots were cultured on media with different PGRs to induce morphogenesis (Fig. 3a). On MS medium with 2.5 mg/L NAA, some yellow callus was induced on root explants. Some adventitious roots formed from the callus within 30 days, but these roots were green and no adventitious shoots or somatic embryos were observed (Fig. 3b).

When white root explants were cultured on MS medium with 2.5 mg/L BA, within 30–60 days, explants became green and swollen (Fig. 3c). Some callus, then protuberances, formed on the surface of explants. Some globular-, heart-, and cotyledon-shaped somatic embryos developed (Fig. 3d–f, respectively).

Fewer adventitious shoots and somatic embryos developed from root explants than from leaf explants at the same concentration of BA or TDZ (Table 3). The highest number of mean 1.1 adventitious shoots and 7.6 somatic embryos on medium with 2.5 mg/L TDZ whereas 2.5 mg/L BA only induced 1.8 adventitious shoots and 4.7 somatic embryos (Table 3).

**Table 3:** Effects of plant growth regulators (PGRs) on the induction of somatic embryos and adventitious shoots from roots of *Scaevola sericea* after light culture for 30 days.

MS + PGRs (mg/L)	Number of adventitious shoots	Number of somatic embryos
NAA 0.1–2.5	0.0 ± 0.0 d	0.0 ± 0.0 e
2,4-D 0.1–2.5	0.0 ± 0.0 d	0.0 ± 0.0 e
BA 0.1	2.9 ± 0.2 ab	0.4 ± 0.3 d
BA 0.5	3.4 ± 0.5 a	2.2 ± 0.4 c
BA 2.5	1.8 ± 0.4 b	4.7 ± 0.4 b
TDZ 0.1	1.3 ± 1.1 bc	2.9 ± 0.9 c
TDZ 0.5	1.3 ± 1.0 bc	4.9 ± 0.3 b
TDZ 2.5	1.1 ± 0.4 c	7.6 ± 0.8 a

Each treatment had 30 leaf explants. Different letters within a column indicate significant differences according to Duncan’s multiple range test (*P* < 0.05).

### Histological observation of somatic embryogenesis from leaf explants

**Figure 4:**
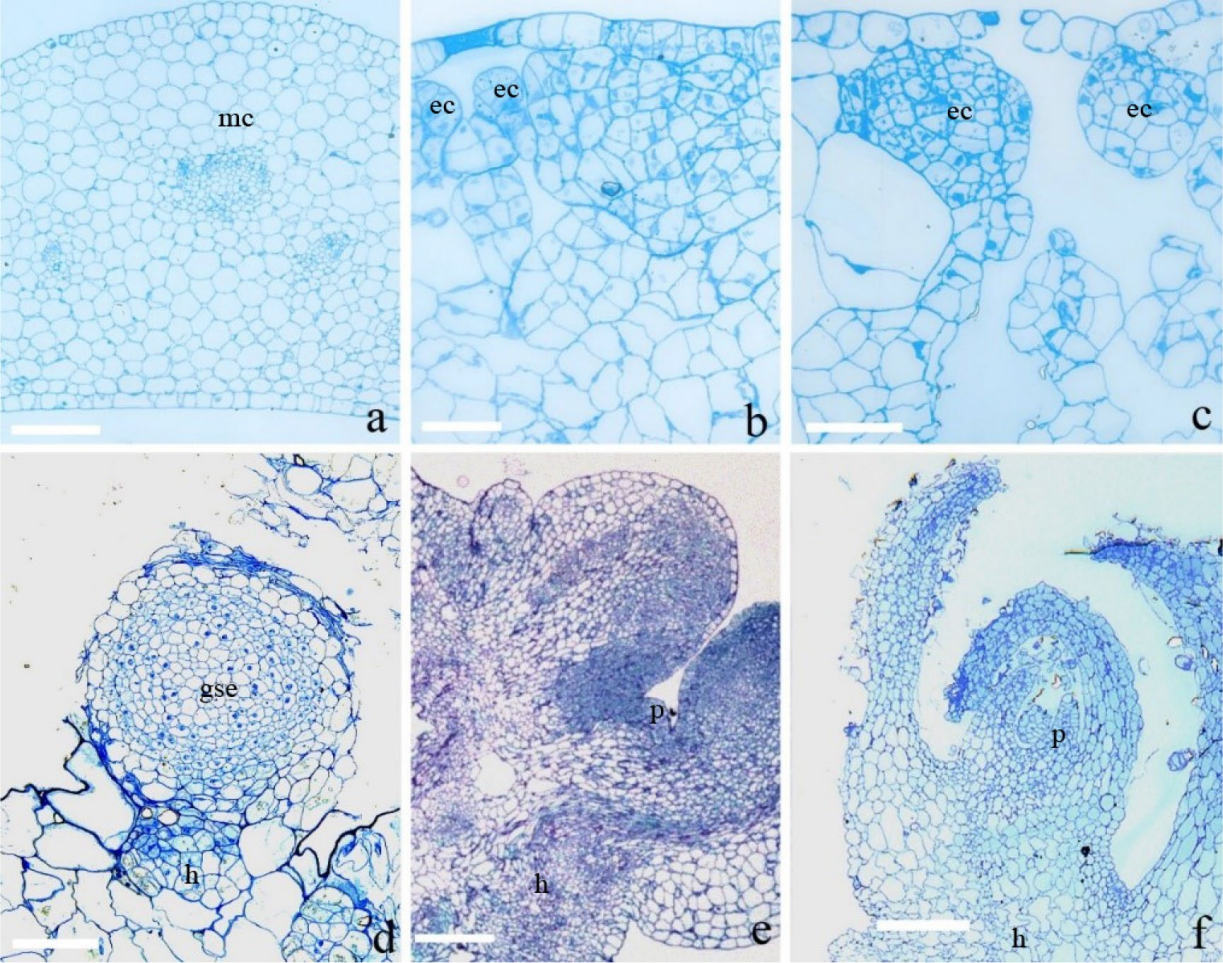
Histological observation of somatic embryogenesis from leaf explants of *Scaevola sericea*. Leaf explants were cultured on MS medium with 2.5 mg/L BA for 0, 10, 20, 30–40, 50, and 60 days (a, b, c, d, e, f, g, respectively). Bars = 0.5 mm; Marked: mc: mesophyll cell; ec: embryogenic cells; gse: a globular somatic embryo; h: hypocotyl, p: plumule. (**a**) fresh leaf section; (**b**) culture for 10 days showing some smaller embryogenic cells; (**c**) culture for 20 days showing some larger embryogenic cells clumps; d, culture for 30–40 days showing a globular somatic embryo with a hypocotyl attached; e, culture for 50 days showing a heart-shaped somatic embryo and plumule meristem development; f, culture for 60 days showing plumule development.

After young leaf explants were cultured on MS with 2.5 mg/L BA, embryogenic callus and somatic embryo development was observed histologically (Fig. 4). Leaves had only one layer of epidermal cells and more than 10 layers of homogeneous parenchyma, with irregular size (Fig. 4a). After culture for 10 days, a few embryogenic clumps formed below the epidermis (Fig. 4b). Culture for more than 20 days resulted in larger embryogenic cells clumps and some globular somatic embryos under the epidermis (Fig. 4c). At this point, the surface of leaf explants was lumpy. After culture for 30 -40 days, globular somatic embryos broke through the epidermis with a suspensor attached (Fig. 4d). After culture for 50 days, globular somatic embryos differentiated into cotyledon-shaped somatic embryos with a new apical meristem (Fig. 4e). After culture for 60 days, the apical meristem developed a new shoot bud and two cotyledons (Fig. 4f).

### Root induction

**Figure 5:**
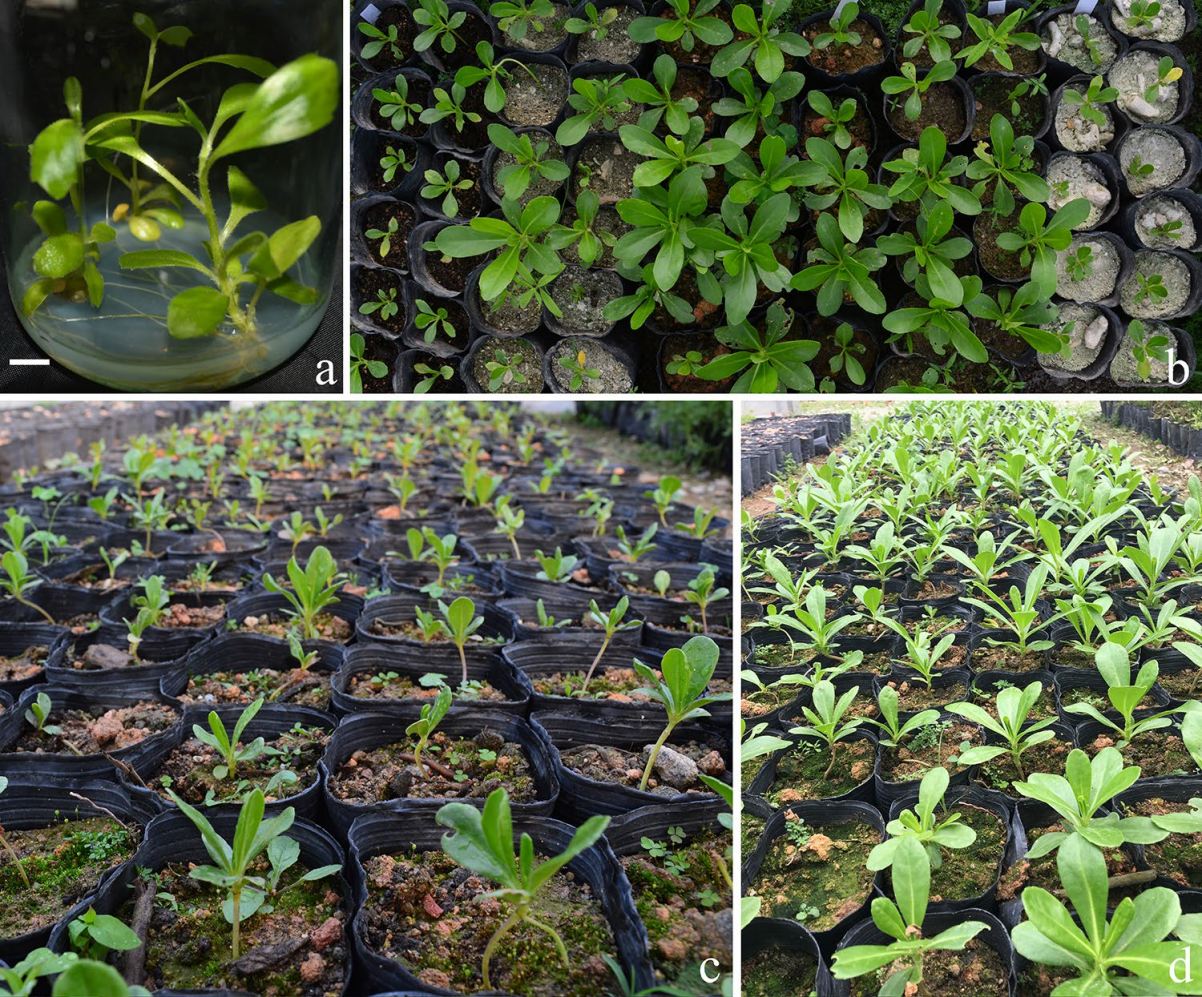
Root formation and transplantation of *Scaevola taccada.* (**a**) Root formation on MS medium with 1.0 mg/L NAA for 45 days (bar = 0.2 cm); (**b**) plantlets were transferred to different substrates for 45 days, showing different growth; (**c**, **d**) a large number of plantlets were transferred to plastic bags with a substrate of yellow mud: peat soil (3:1, v/v), which was the optimal substrate (Table 5), and left to grow for one and three months, respectively.

Only 10% of shoots developed roots within 30 days on PRG-free 1/2 MS medium. However, any auxin induced a high percentage of rooting (> 81%) within 30 days (Table 4, Fig. 5a)*.* Higher concentrations of NAA or IBA induced more roots, such as 0.1 mg/L NAA induced 81% rooting and 2.5 mg/L NAA induced 99% rooting.

**Table 4:** Effect of plant growth regulators (PGRs) on the rooting of *Scaevola sericea* shoots after culture for 30 days.

1/2MS + PGRs (mg/L)	Rooting percentage (%)
CK	10.1 ± 4.4 d
IBA 0.1	91.9 ± 4.9 b
IBA 0.5	93.3 ± 4.4 ab
IBA 2.5	98.8 ± 1.2 a
NAA 0.1	81.6 ± 1.1 c
NAA 0.5	86.5 ± 3.9 bc
NAA 2.5	99.0 ± 1.0 a
IAA 0.1	83.5 ± 4.9 c
IAA 0.5	83.8 ± 3.7 c
IAA 2.5	86.5 ± 1.9 bc
IBA 1.0 + NAA 1.0	93.9 ± 3.4 ab
IBA 1.0 + NAA 0.2	94.9 ± 3.2 ab

Each treatment had 30 shoots. Different letters within a column indicate significant differences according to Duncan’s multiple range test (*P* < 0.05).

### Acclimatization and transplanting

When plantlets were transplanted to different rooting substrates for 45 days, 91.7% of plantlets survived in river sand and coral sand (1:1). In coral sand, only 66.8% survived (Table 5, Fig. 5b). In the substrate yellow mud: peat soil (3:1) and river sand, survival percentage was only 73–74%, but plantlets were larger and taller (Table 5, Fig. 5c,d).

**Table 5:** Effects of substrates on the survival of transplanted *Scaevola sericea* plantlets after transplanting for 30 days.

Substrates (volumetric ratios)	Survival (%)	Average increment of shoot length (cm)
Yellow mud: peat soil (3:1)	73.5 ± 1.5 c	4.8 ± 0.3 a
River sand	74.5 ± 0.5 c	3.6 ± 0.6 b
River sand: coral sand (1:1)	91.7 ± 2.7 a	1.8 ± 0.3 c
Peat soil: vermiculite: perlite (3:2:1)	82.7 ± 0.35 b	1.9 ± 0.4 c
Peat soil: vermiculite: perlite (1:1:1)	73.2 ± 1.8 c	1.1 ± 0.2 d
Coral sand	66.8 ± 3.2 d	1.1 ± 0.3 d

Each treatment had 30 plantlets. Different letters within a column indicate significant differences according to Duncan’s multiple range test (*P* < 0.05).

## Discussion

A number of commercially available cultivars or species of the genus *Scaevola*, including *S. aemula*, *S. albida*, *S. phlebopetal*, and *S. striata*, as well as *S. glandulifera*, *S. hookeri* and *S. ramonissima* growing wild, were successfully shoot propagated by tissue culture^15,16^.

Plant regeneration from callus of fan flower, *S. phlebopetala*, was also achieved. The effects of KIN, 2,4-D and BA on callus and shoot induction were investigated. KIN was not required for callus induction, 2,4-D induced poor quality callus while BA had a significantly positive effect on callus induction^17^.

On MS medium with 2.0 mg/L BA and 5.0% coconut juice, *S. aemula* induced axillary shoots from stem segments although neither of these supplements was essential for shoot induction^18^. The leaf explants of *S. aemula* induced somatic embryos on MS medium with 0.2 mg/L 2,4-D and 0.2–0.5 mg/L BA. When globular somatic embryos were transferred to MS medium with 0.5 mg/L BA, the frequency of conversion to plantlets was high^15^. When *S. aemula* leaf explants were cultured on MS medium with different concentrations and combinations of BA and NAA, they could induce adventitious shoots and roots^18^. Wang and Bhalla^19^ reported that *S. aemula* leaf and root explants were used to induce callus in a suspension culture on MS medium supplemented with 0.2 mg/L 2,4-D and 0.5 mg/L BA in darkness. After 6 weeks, cells in suspension culture were transferred to solidified callus medium and cultured in the dark forming microcallus 2 weeks after transfer to regeneration medium in the light. Shoot regeneration was 19.2% and 13.9% from leaf- and root-derived callus, respectively^19^. In a study by Wang, direct differentiation of embryogenic structures was first reported in mesophyll protoplast cultures of *S. aemula*^20^. The isolated protoplasts initiated cell division when cultured in MS medium and higher plating efficiencies were observed in medium with a combination of NAA and BA than with 2,4-D and BA, ultimately forming globular somatic embryos. Direct and indirect somatic embryogenesis from petiole and leaf explants of *S. aemula* cv. ‘Purple Fanfare’ was achieved^21^. A high frequency of somatic embryos was obtained directly from petiole and leaf explants using TDZ. Petiole explants formed more somatic embryos than leaves, plants derived from somatic embryos of petiole explants germinated more readily into plants, and somatic embryos formed more efficiently on 1/2 MS medium than in full-strength MS medium^21^.

In our paper, the leaves and roots of *S. sericea* were used as explants and cultured on MS medium supplemented with BA, TDZ alone or BA combined with NAA, inducing both adventitious shoots and somatic embryos but 2,4-D and NAA could not induce either organs directly, indicating that only cytokinins can induce both organs. These results are similar to other species such as the rare and endangered species *Metabriggsia ovalifolia* W. T. Wang^22,23^ and *Primulina tabacum* Hance^24,25^. Leaf explants of *P. tabacum* only induced somatic embryos if cultured continuously on TDZ-supplemented medium, but if cultures were transferred to medium with BA, both somatic embryos and adventitious shoots could be induced^25^. BA induced more adventitious shoots from leaf explants than TDZ while TDZ induced more somatic embryos than BA from root explants. In *S. sericea*, embryogenic cells originated from inner mesophyll cells and not from epidermal cells, very different from *P. tabacum* where embryogenic cells originated from both the mesophyll and epidermal cells^25^.

Roots have been used as explants to induce shoots in a some plant species. Shoot buds were induced from roots of *Citrus mitis* Blanco seedlings when cultured on the MS medium supplemented with 2.2 µM BA^26^. Shoot buds were differentiated from the pericycle at the proximal region of *Citrus aurantifolia* Christm roots on the MS medium supplemented with 2.2 µM BA^27^. Shoot buds were also formed on the proximal end of root of *Lotus corniculatus* L on MS medium supplemented with 0.44 μM BA^28^. In *Spinacia oleracea* L., shoots were induced from the central root region on MS medium supplemented with 20 μM NAA and 5.0 μM gibberellic acid^29^. The optimum conditions for regeneration from leaf, stem and root explants of *Gentiana triflora* cv. WSP-3 were the MS medium supplemented with 5–10 mg/L TDZ and 0.1 mg/L NAA for the leaf and stem explants, and 10 mg/L TDZ and 1 mg/L NAA for the root explants^30^. The root explants of *Ocimum basilicum* L. cultured on the MS medium supplemented with 0.1–10 μM BA or TDZ induced shoot buds, TDZ could induce shoots at 100%, which was much more than that of BA^31^. The effect of various levels of BA and TDZ on the shoot bud regeneration from root explants of *Populus tremula* L. was studied, TDZ in the medium stimulated a tenfold increase in the number of shoot buds than BA from root explants. However, it reduced further shoot development and elongation. Root biomass production and growth was greatly enhanced in a well-aerated bioreactor in the medium supplemented with 4.5 × 10^–2^ μM TDZ^32^. The root explants of *Aralia elata* cultured on the broad-leaved tree medium with 1.0 μM BA could induce means 18 adventitious shoot buds/root explant^33^. An efficient protocol for shoot regeneration from root explants was developed for *Centaurea ultreiae*, a critically endangered species. Organogenesis from root explants was promoted by incubating the root explants on 1/2 MS medium supplemented with one of four cytokinins and the best response (94.3%) of the root explants was in medium supplemented with 0.55 µM BA, producing means 5.6 shoots per explant^34^. About 14–21 adventitious shoots per root explant formed on the roots of semi-parasitic plant *Monochasma savatieri* Franch ex Maxim by supplementing 2.5 µM of three cytokinins (KIN, BA and TDZ) in MS medium^35^. The roots became intense green and swollen in all of the IMs tried, except for the medium containing 0.2 mg /l BA after 2 weeks of culture. The initiation of shoot-bud induction was longer and need 4 weeks from root explants. However, the others explant types required only 2–3 weeks^36^. Anatomical events and ultrastructural aspects of direct and indirect shoot organogenesis in *Passiflora edulis* were studied, as root explants were cultured on the MS induction medium supplemented with 1.0 mg/L BA. During direct organogenesis, the adventitious buds and nodules, formed from meristemoids, originated from the pericycle regions distant from the cut surface. Completely differentiated buds were observed within 20 days. During indirect shoot organogenesis, shoot buds formed on the calli via meristemoids at the periphery, which differentiated from the cortical region of the initial explant^37^. *Passiflora cincinnata* Mast and 0.5 mg/L 6-BA in MS medium was most suitable for shoot regeneration from the root explants^38^. A framework for shoot regeneration from *Arabidopsis* root explants in vitro have been build. It was approved that regenerated shoots was originated directly or indirectly from the pericycle cells, which was adjacent to xylem poles which indicated the pericycle is an extended meristem that comprised two types of cell populations^39^.

Root explants could induce somatic embryogenesis although this plant regeneration pathway were not so popular. Plants were regenerated from adventitious roots of rose (*Rosa hybrida*) rootstock 'Moneyway' via an indirectly three-step procedures: callus induction, induction of somatic embryos and shoot development^40^. The somatic embryos were induced from apical sections of seedlings (1 cm) lateral roots of *Spinacia oleracea* L., which were cultivated on the MS medium supplemented with 20 µM NAA and 5 µM GA_3_. It had no obvious calli formation and all types of somatic embryos were visible within 12 weeks ^41^. The research verified that the effect of genotype on regeneration capacity showed a significantly better response in explants exposed to long day (16-h photoperiod) than to short day (8-h photoperiod)^42^. Somatic embryogenesis and shoot organogenesis from root explants of Lisianthus (*Eustoma grandiflorum*) could be alternatively induced by auxin or cytokinin. Somatic embryonic calli was induced in the dark on the MS medium supplemented with 10 μM 2,4-D and then the somatic embryonic calli were transferred to an embryo conversion phase medium containing 2 μM BA to promote full plantlet development^43^.

In our study, roots of *S. sericea* were used as explants to induce successfully somatic embryogenesis directly. Among the cytokinins employed, TDZ induced more somatic embryos than BA. This was the first report on direct somatic embryogenesis without any obvious calli formation from in vitro root- derived explants of *S. sericea*.

As a semi-mangrove plant, *S. sericea* has certain requirements for permeability and salinity in transplanting substrates. Transplanting had the highest survival percentage in the substrate of 100% sand or river sand mixed with coral sand, although the increase in plant height and leaf expansion were poor after 45 days (Table 5). The transplanting survival percentage was low in the substrate with peat and loess because of root decay which led to plant death. This is because peat soil is rich in humus, providing sufficient nutrition for plant growth but also high bioaccumulation, rich in bacteria, and less hydrophobic than sand, giving a rotten appearance to roots.

## Conclusions

An efficient regeneration system via shoot organogenesis and somatic embryogenesis from in vitro leaf and root explants was established for  *S. sericea* for the first time. BA and TDZ, could induce adventitious shoots and somatic embryos from leaf and root explants. Neither 2,4-D nor NAA were able to directly induce adventitious shoot or somatic embryo. Maximum rooting percentage (98–99.0%) was achieved on half-strength MS medium supplemented with 2.5 mg/L IBA or NAA. Well-rooted plantlets, which were transplanted into a substrate of pure river sand, displayed a high survival percentage of 91.7% after transplanting for 45 days while the best substrate for plantlet growth was River sand: coral sand (1:1).

## Methods

Mother plants of *S. sericea* were collected from Wenchang City, Hainan Province, China and introduced to the propagation base of South China Botanical Garden, in Guangzhou, China. Young stem segments with axillary buds (3–4 cm long) of 2-year-old mother plants were used as explants. A trial was conducted using Murashige and Skoog (MS) or 1/2 MS (half the level of MS macro nutrients) medium^44^ containing 3% sucrose, 0.7% agar (Solarbio, Beijing) and pH 5.8 and different types and concentrations of plant growth regulators (PGRs) (Sigma-Aldrich, St. Louis, MI, USA). Thidiazuron (TDZ) was added post-sterilization after filtering (0.24 µm) while all other PGRs were added directly to media sterilized at 121 °C and 104 k Pa for 18 min. Cultures were placed in a culture room illuminated by two cool white fluorescent lamps (40 W each; Philips, Tianjing, China) at a photon flux density of 80 µmol m^-2^ s^-1^ in a 12-h photoperiod in a 25 ± 1 °C culture room. Stem sections 1.5–3 cm long from healthy branches of mother plants were cut, leaves were removed, and denuded stem sections were soaked in 0.1% HgCl_2_ for 8 min on an ultra-clean workbench then rinsed in sterile distilled water seven times. Rinsed sections were dried on sterile filter paper and inoculated onto MS medium supplemented with 0.5 mg/L 6-benzyladenine (BA) and 0.1 mg/L NAA to induce axillary shoots using a protocol that was employed for six halophytic species, including *S. sericeay*^14^.

### Proliferation of axillary shoots

Axillary shoot clumps were cut into smaller clumps with 3–4 shoots and inoculated onto MS media supplemented with different combinations and concentrations of PGRs for axillary shoot proliferation (Table 1). In each treatment, 10 jars were inoculated and each jar contained five shoot clumps. After culture for 40 days, the axillary shoot proliferation coefficient (SPC) was assessed as: number of axillary shoots after proliferation for 45 days / the number of axillary shoots before proliferation.

### Induction of adventitious shoots and somatic embryos from leaf explants

Young in vitro leaves from axillary shoot proliferation on MS medium supplemented with 1.0 mg/L BA and 0.1 mg/L NAA were cut into smaller pieces (about 1.0 × 1.0 cm) then inoculated onto MS medium supplemented with different types and concentrations of PGRs and their combinations (Table 2, Fig. 1a). In each treatment, 10 jars were inoculated and each jar contained five leaf explants. After light or dark culture for 40 days, respectively, induction of callus, the number of adventitious shoots and somatic embryos was assessed based on their shapes and appearance.

### Induction of shoots and somatic embryos from root explants

Shoots cultured on MS medium supplemented with 0.5 mg/L NAA for one month developed some new adventitious roots. These roots were cut into 1.0 cm long segments, including the root tip, and cultured on different MS media in the light (Table 3, Fig. 3a). In each treatment, 10 jars were inoculated and each jar contained five root explants. After culture for 40 days, the number of adventitious shoots and somatic embryos was assessed based on their shape and appearance under a microscope (BDS200, OPTEC, Chongqing, China).

### Histological observation

Young leaf explants were cut into small segments (0.5 cm in size) and then cultured on MS medium supplemented with 2.5 mg/L BA for 0, 10, 20, 30, 40, 50, or 60 days. Explants were removed from culture jars and fixed immediately in 2.5% paraformaldehyde (Fluka, Buchs, Switzerland) and 2.5% isovaleraldehyde (Sigma-Aldrich) in 0.1 M sodium phosphate buffer solution (pH 7.2) at room temperature for 24 h, then vacuum-infiltrated for 1 day at 4 °C. Explants were dehydrated in 30%, 50% and 70% ethanol for 20 min each, then dehydrated in 80% and 90% ethanol for 15 min each at room temperature. Leaf explants were transferred to 100% ethanol twice for 30 min each time, and then twice in pure propylene oxide (Seebio, Shanghai) for 30 min each time. Explants were soaked for 30 min in a mixture of epoxy propane and resin (EPON812) (Wirsam, Cape Town, South Africa) (3:1, v/v), then in a mixture of epoxy propane and resin (1:1) for 1 h, and finally for 2 h in a mixture of epoxy propane and resin (1:3, v/v). Explants were infiltrated with pure resin at room temperature and baked in an embedding box at 40 °C for 7 days, sliced in 2 µm sections, then stained with toluene blue. Sections were observed under microscope (Olympus SZX12).

### Root induction

Axillary shoots were cut into single shoots which were cultured on different rooting media supplemented with different concentrations and combinations of IBA and NAA (Table 4). In each treatment, 10 jars were inoculated and each jar contained three shoots for rooting. PGR-free 1/2MS medium served as the control. After 40 days of culture in light, rooting percentage was assessed: (number of buds that rooted after 40 days / number of inoculated buds) × 100%.

### Acclimatization and transplantation

Culture jars with shoots were cultured on MS rooting medium supplemented with 2.5 mg/l IBA for 30 days in a culture room then transferred to natural light conditions for 7 days. Agar was gently rinsed off roots under tap water. Rooted plantlets were transplanted into the following substrates (v/v): (1) yellow mud / peat soil (3:1); (2) 100% river sand; (3) river sand / coral sand (1:1); (4) peat soil / vermiculite / perlite (3:2:1); (5) peat soil / vermiculite / perlite (1:1:1); (6) 100% coral sand. All the substrates were filled into black plastic nursery bags (12 cm high and 10 cm in diameter), and water was sprayed on the leaf surface every morning and evening. Each treatment had 30 plantlets and every bag contained only one plantlet. After 45 days, plantlet height and survival percentage of transplanted plantlets were determined: (number of living plantlets before transplanting / number of living plantlets after transplanted) × 100%. Remaining plantlets were transferred to the best growth substrate for scaling-up cultivation.

### Data and statistical analysis

Experimental data, all in triplicate and with 30–50 samples treatment, were statistically processed using SPSS 17.0 software (IBM). Data are represented by mean ± standard error. Duncan’s multiple range test was used to assess significant differences between means at *P* < 0.05.

### Ethics approval and consent to participate

Not applicable.

### Consent for publication

Not applicable.

## Data Availability

All data generated or analyzed during this study are included in this published article and its supplementary information files.

## Abbreviation

MS: Murashige and Skoog medium
2,4-D: 2,4-Dichlorophenoxyacetic acid
NAA: *α*-Naphthaleneacetic acid
TDZ: Thidiazuron
BA: 6-Benzyladenine
KIN: Cytokine
PGRs: Plant growth regulators
